# FUSIM: a software tool for simulating fusion transcripts

**DOI:** 10.1186/1471-2105-14-13

**Published:** 2013-01-16

**Authors:** Andrew E Bruno, Jeffrey C Miecznikowski, Maochun Qin, Jianmin Wang, Song Liu

**Affiliations:** 1 Department of Biostatistics, SUNY at Buffalo, , Buffalo, NY 14214, USA; 2 Department of Biostatistics and Bioinformatics, Roswell Park Cancer Institute, Buffalo, NY, 14263, USA; 3 Center for Computational Research, SUNY at Buffalo, , Buffalo, NY, 14260, USA

## Abstract

**Background:**

Gene fusions are the result of chromosomal aberrations and encode chimeric RNA (fusion transcripts) that play an important role in cancer genesis. Recent advances in high throughput transcriptome sequencing have given rise to computational methods for new fusion discovery. The ability to simulate fusion transcripts is essential for testing and improving those tools.

**Results:**

To facilitate this need, we developed FUSIM (FUsion SIMulator), a software tool for simulating fusion transcripts. The simulation of events known to create fusion genes and their resulting chimeric proteins is supported, including inter-chromosome translocation, trans-splicing, complex chromosomal rearrangements, and transcriptional read through events.

**Conclusions:**

FUSIM provides the ability to assemble a dataset of fusion transcripts useful for testing and benchmarking applications in fusion gene discovery.

## Background

Chromosome aberrations and their corresponding gene fusions play an important role in carcinogenesis and cancer morbidity [1]. The identification of fusion genes such as TMPRSS2-ERG [2], EML4-ALK [3], and BCR-ABL1 [4], have led to successful diagnostic biomarkers and therapeutic targets. Thus methods for detecting fusion genes and their corresponding chimeric proteins have major clinical significance. Recent advances in next-generation sequencing (NGS) and high-throughput transcriptome sequencing (RNA-Seq) have paved the way for new methods in fusion gene discovery. One of the major challenges in identifying novel fusion transcripts is controlling the high false positive rate. The majority of methods in recent publications utilizing RNA-Seq data [5-10], employ advanced filtering steps to eliminate false positives and nominate a set of potential fusion candidates. However, fusion validation involves a substantial amount of manual effort requiring the design of complex PCR primers which can significantly drive up costs. As a result, only a portion of predicted fusion events subject to experimental validation. Measuring the accuracy of these methods is becoming increasingly important to help improve future algorithm development. To help facilitate this need, we developed FUSIM, a software tool for simulating fusion transcripts from gene models. An advanced set of features are available for controlling fusion transcript simulation modeled after characteristics of gene fusions in vivo. FUSIM enables comprehensive testing *in silico* of fusion discovery methods in transcriptome sequencing data. FUSIM is open source software written in Java and runs on any platform supporting Java version 1.6 and above.

## Implementation

### Input

FUSIM requires as input, the number of fusion transcripts to generate and a gene model file in UCSC GenePred table format [11]. General Feature Format (GFF [12]) and Gene Transfer Format (GTF [13]) files are also supported using FUSIM’s built in GTF-to-genePred converter. FUSIM also requires an faidx-indexed reference genome file for use in outputting raw fusion sequences. Reference genomes in FASTA format [14] can be converted to faidx-indexed format using SAMtools [15]. FUSIM can optionally simulate fusion transcripts based on the expression levels of genes found in experimental data. If this option is selected, a file of RNA-Seq read alignments in Binary Alignment/Map (BAM [15]) format is required.

### Gene selection

FUSIM supports two modes for selecting genes to be included in fusion transcripts. The first mode (default) randomly selects genes from the provided gene model using a discrete uniform distribution where each gene has equal weight $\frac{1}{n}$. The second mode selects genes using a background dataset of RNA-Seq read alignments in BAM format. The background RNA-Seq dataset is first preprocessed using the provided gene model and for each gene, the reads per kilobase of exon model per million mapped reads (RPKM [16]) is computed. A default RPKM cutoff of 0.2 is used to filter out genes with low expression and can be optionally configured by the user. Genes are then selected using one of the three methods: *uniform*, *empirical*, or *binned*. The *uniform* method simply selects genes at random having an RPKM value above the cutoff. The *empirical* method randomly selects genes based on the empirical distribution of RPKM values in the background RNA-Seq dataset. The empirical distribution is a non-parametric estimation of the probability distribution function of RPKM values. Our (histogram) estimator is a piecewise constant function where the height of the function is proportional to the number of observations in each bin. The number of bins is a smoothing parameter and can be chosen according to many rules. For simplicity, we choose $k\;=\;\sqrt{n}$ where *n* is the number of genes (RPKM values). Alternatively, FUSIM also offers the Sturges’ method where *k*=log_2_*n*+1. The *binned* method sorts genes into *m* bins using their RPKM values, where *m* is the number of fusions to generate. A set of genes are then selected once from each bin, covering the dynamic range of gene expression contained in the background RNA-Seq dataset. The gene selection modes along with their corresponding options in FUSIM are summarized in Table 1.

**Table 1:** Gene selection options in FUSIM

**Mode**	**Method**	**Options**	**Description**
Random (default)	uniform	‐l,‐limit	Limit all fusions to specific geneId, transcriptId, or chrom
		‐1,‐gene1	Filter for gene1
		‐2,‐gene2	Filter for gene2
		‐3,‐gene3	Filter for gene3
Background	uniform emprical binned	‐b,‐background‐reads	Path to BAM file containing background reads. Genes will be selected for fusions according to the read profile of the background reads
		‐k,‐rpkm‐cutoff	RPKM cutoff when using background BAM file. Genes below the cutoff will be ignored
		‐m,‐gene‐selection‐method	Method to use when selecting genes for fusions uniform|empirical|binned
		‐p,‐threads	Number of threads to spawn when processing background BAM file

Gene selection modes and corresponding options in FUSIM.

The filters to select genes by gene ID, transcript ID, or chromosome are also supported. They can be set globally (i.e. specifying all genes within a fusion) or set on a per gene basis. For example, specifying only the first gene in a fusion to BCR or both the first and second gene to BCR and ABL1 respectively. This provides the ability to specify simulation of fusion transcripts on genes or chromosomes of interest.

### Types of fusions

**Figure 1:**
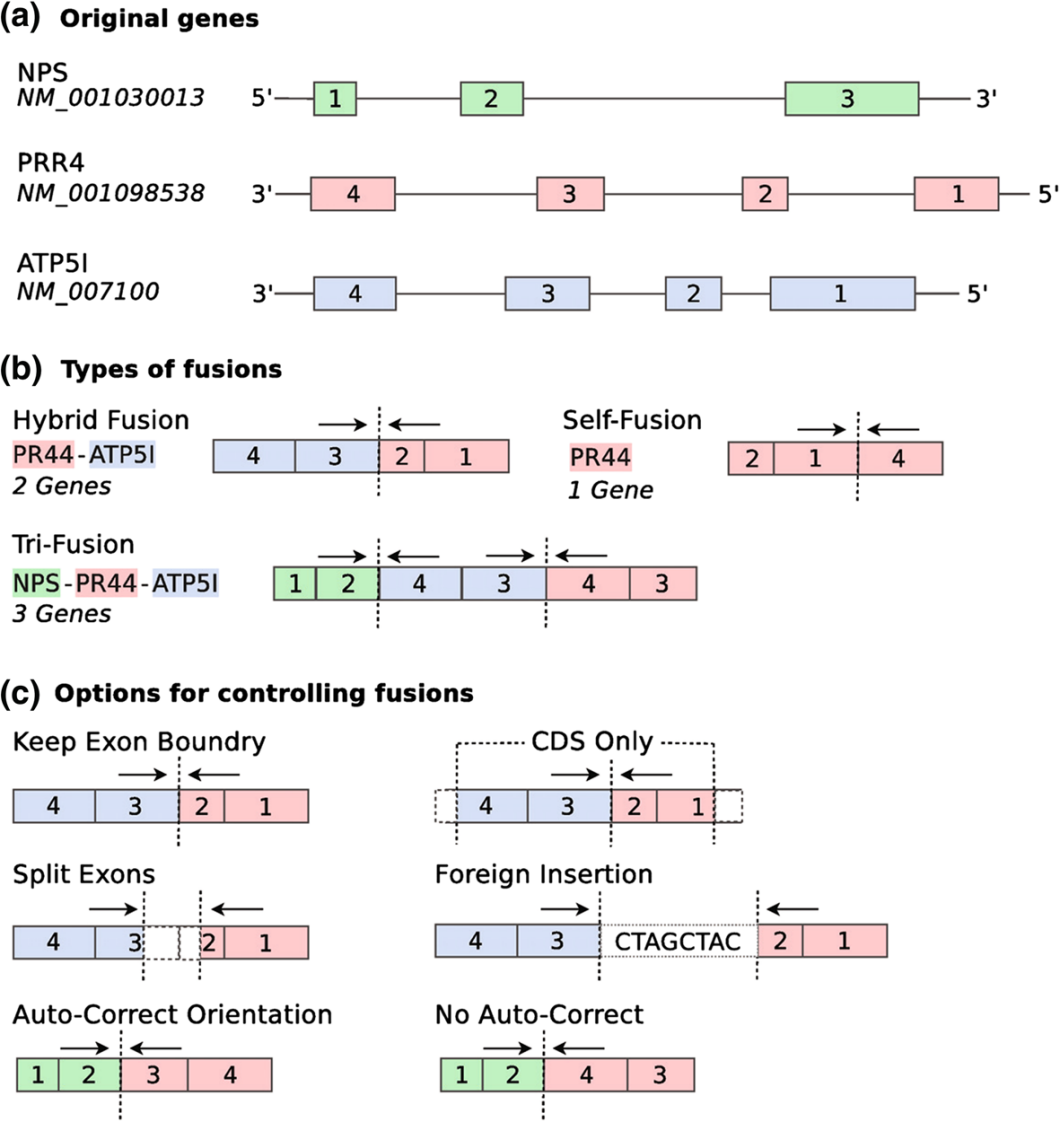
**Fusion transcript simulation. **Example of fusion transcript simulation. **(a)** Original transcripts of three selected genes NPS, PR44, ATP5I. Boxes represent exons and solid lines refer to introns. **(b) **Illustration of three basic types of fusion transcripts. *Hybrid fusions *use exons from two distinct genes, *Self fusions* join exons from a single gene, *Complex fusions *use exons from three distinct genes. **(c) **Example of the available options for controlling fusion transcript generation. *Split exons *randomly selects breakpoints in the exons involved. *Keep exon boundary *forces fusion breakpoints to fall on exon boundaries. *CDS only *creates fusions using exons within the coding sequence region. *Foreign insertion *inserts a randomly generated sequence between fusion breakpoints. *Auto-correct orientation *forces FUSIM to correct the orientation of exons.

Multiple types of fusion transcripts are supported in FUSIM. Based on the number of genes involved per fusion, the fusion types are classified as self, hybrid, complex which involves 1, 2, and 3 genes respectively (Figure 1b). According to chromosome locations and strand, fusion types are classified as CTX (inter-chromosome events), ITX (intra-chromsome events with different strands), DUP (tandem duplication), and DEL (deletion). Complex chromosomal rearrangements (CCRs) are examples of complex fusions involving at least three breakpoints on two or more chromosomes [17]. Read through events are also supported as a special case of DEL with two adjacent genes on the same strand fused together. The distribution of known gene fusion events in various tissues and/or diseases can be derived from online databases such as ChimerDB [18]. For example, the proportion of CTX, ITX, DUP and DEL in all known cancer fusion events is 88.3%, 4.8%, 5.5% and 1.4%, respectively. FUSIM enables users to create simulated datasets based on the distribution of known fusion catalogs in the specific tissue/disease type of their study (if available).

### Options for controlling fusions

After selecting a set of genes using the methods outlined in the previous section, fusion transcripts are created by randomly choosing a breakpoint in each gene and fusing them together. Breakpoints are created by randomly selecting *n* number of consecutive exons from the start or end of each gene.

FUSIM provides an advanced set of options to further control various aspects of fusion transcript simulation (Figure 1c). By default, genes are fused together by splitting the joined exons in random positions (split exons). The keep exon boundary option will fuse genes exclusively on exon boundaries. The CDS only option creates fusions using exons within the coding sequence region, by default all exons are considered. The foreign insertion option inserts a randomly generated sequence between the fusion breakpoint. FUSIM can be set to auto-correct the orientation of the resulting fusion transcript if genes are located on different strands. This is done by reverse complementing the selected exons to match the orientation of the first gene in the fusion. By default, FUSIM creates in-frame fusion transcripts preserving the reading frame. Generating out-of-frame fusion transcripts disrupting the reading frame is also supported.

### Output

**Figure 2:**
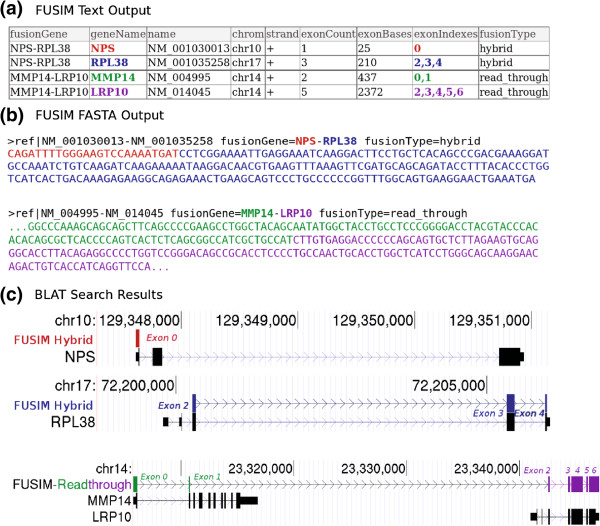
**Example FUSIM output. **Example of simulated fusion transcripts generated by FUSIM. **(a) **Text output of two simulated fusion transcripts NPS-RPL38 (inter-chromosome hybrid fusion) and MMP14-LRP10 (readthrough fusion). **(b) **FASTA output of the raw sequence data showing fusion junctions. **(c) **Results of BLAT search using the FASTA sequences in (b) validating FUSIM output. The black square boxes represent the exons from RefSeq genes and the colored boxes represent the exons from the gene fusion generated by FUSIM.

FUSIM outputs simulated fusion transcripts in both plain text and FASTA format as shown in Figure 2. The text output format used by FUSIM is very similar to UCSC GenePred (refFlat) format and can be easily parsed with exiting software.

Certain fusion discovery tools require sequencing read data in FASTQ [19] format as input. FUSIM includes wrapper scripts for simulating next generation sequencing reads from the generated fusion transcripts using ART [20]. The resulting FASTQ files can also be aligned back to a reference genome and optionally merged with existing alignment data, useful for injecting reads from simulated fusions into background datasets.

## Conclusion

One of the main difficulties in testing fusion discovery methods is the lack of a golden standard dataset of fusion transcripts which can be used to accurately compare performance. FUSIM aims to provide a convenient way to rapidly generate datasets of simulated fusion transcripts for comprehensive comparison across fusion discovery methods. The advanced options in FUSIM allow for construction of simulated fusion transcripts that model the origins of gene fusions *in vivo*.

## Availability and requirements

**Project name:** FUSIM**Project home page:**http://aebruno.github.com/fusim/**Operating system(s):** Platform independent**Programming language:** Java**Other requirements:** Java 1.6 or higher**License:** Apache License version 2.0**Any restrictions to use by non-academics:** none

## Abbreviations

GenePred: Gene Prediction Track Format; GFF: General Feature Format; GTF: Gene Transfer Format; BAM: Binary Alignment/Map; RPKM: reads per kilobase of exon model per million mapped reads.

## Competing interests

The authors declare that they have no competing interests.

## Authors’ contributions

AEB designed and implemented the software. AEB also drafted the manuscript. SL and JW conceived of the project. SL, JW, MQ, and JCM provided feedback on the software development and manuscript. AEB, JCM, JW, MQ, and SL tested the software. All authors read and approved the final manuscript.
